# Buckling Structured Stretchable Pseudocapacitor Yarn

**DOI:** 10.1038/s41598-017-12375-7

**Published:** 2017-09-20

**Authors:** Duck Weon Lee, Jung Han Lee, Nam Ki Min, Joon-Hyung Jin

**Affiliations:** 1 Department of Ray H. Baughman Lab, Jiangnan graphene research institute, No.6 XiangYun Road, Wujin Economical Development Zone, Jiangsu, 213149 China; 2 Nanomedicinal research laboratory, Inha university school of medicine, Jeongserk Bldg.A, Seohae-daero 366, Jung-gu, Incheon, 22332 Korea; 3 Department of Control and Instrumentation Engineering, Korea University, Sejong-ro 2511, Sejong, 30019 Korea; 4 Department of Chemical Engineering, Kyonggi University, 154-42 Gwanggyosan-ro, Yeongtong-gu, Suwon-si, Gyeonggi-do, 16227 Korea

## Abstract

Cable-type stretchable electrochemical pseudocapacitors based on multi-walled carbon nanotube (MWCNT) sheets and two different metal oxide nanopowders (NP), i.e., MnO_2_ and RuO_2_ are developed using a newly-devised dry painting method to mechanically fix the NP to the elastic rubber-based MWCNT electrode substrate, resulting in a porous buckling structured pseudocapacitor yarn. Highly stretchable stylene-ethylene/butylene-stylene (SEBS) is used as the supporting elastomeric core for wrapping with the MWCNT sheets and the electroactive NP. The dry painting can successfully deposit NP on the soft SEBS surface, which is normally an unfavorable substrate for coating alien materials. The resulting yarn-type pseudocapacitor, composed of eight-layered MWCNT sheets, three-layered RuO_2_, and two-layered MnO_2_, showing a diameter of approximately 400 μm with a porous buckling structure, records a specific capacitance of 25 F g^−1^. After being stretched by 200% in strain with no sacrifice of the porous buckling structure, the cable-type stretchable electrochemical pseudocapacitor yarn retains its electrical capacity, and is potentially applicable to energy storage devices for wearable electronics.

## Introduction

Electrochemical capacitors are of great interest to a variety of industrial fields that demand high power density, such as devices that range from small electronic hand-held tools to relatively large electric vehicles, including rails and trams. They all commonly require ultrahigh electrochemical energy storage devices, and the field is even expanding to personal energy-carrying equipment in the form of wearable electronics^1^. Indeed, stretchable and wearable fabrics are about to be realized by the development of stretchable yarn- and sheet-type supercapacitors, which are also applicable to military purposes^2–9^. Such high energy storage devices are frequently combined with secondary batteries^10,11^, energy harvesting devices including photoelectrochemical cells, and finally, integrated power packs^12,13^.

Energetically, pseudocapacitors have more potential when charging a device with high energy storage capacity, due to the electrochemical capacitor-specific double-layer structure, and additional faradaic contribution from redox active materials. They are mostly prepared from transition metal oxides, metal sulfides, and in many cases, carbonaceous metal hybrid compounds^14–21^. In addition to the excellent cyclability, these materials have multiple redox potentials, enabling more rectangular-shaped current-voltage characteristics. As a result, high specific capacity with sufficient robustness can be obtained. However, the physically strong and age-resistive inorganic transition metals are expensive and inherently rigid, which makes them incompatible with stretchable devices in spite of the fact that some flexible devices based on nanowired materials have been introduced^22–24^. As an alternative, organic polymeric conductor-based pseudocapacitors are relatively inexpensive. The conducting polymer layers are easy to prepare via anodical electropolymerization, or spin coating followed by heat or UV curing^25^. Best of all, such conducting polymers are basically excellent bendable and stretchable materials. A common problem is that the organic material-based electrochemical capacitors suffer from limited cycling stability, and unwanted leakage current that mainly depends on the equivalent series resistance. Upon repeated charging and discharging processes, mechanically weak conducting polymers employed for a yarn-type pseudocapacitor can be more susceptible to time-dependent aging^26^. Supercapacitor yarns sandwiched in between two stretchable plastic substrates for better stability and stretchability have been reported^27,28^.

In this work, we introduce a newly devised highly versatile dry painting method to prepare metal oxide-based stretchable pseudocapacitors that have a porous buckling structure. As compared with the conventional electrochemical plating for metal oxide deposition, which usually produces a fragile metallic thin film in a liquid electrolyte phase, the dry painting method employs metal oxide nanopowders (NP) such as RuO_2_ and MnO_2_, and physically deposits the NP on a multi-walled carbon nanotube (MWCNT)-layered stretchable stylene-ethylene/butylene-stylene (SEBS) substrate in atmospheric condition. The physicochemical properties of the highly stretchable SEBS have been discussed elsewhere^29^. The RuO_2_ and MnO_2_ layers are dry painted on the substrate by turn, and the densified MWCNT sheet layer between them effectively holds the NP tightly on the substrate. The resulting stretchable pseudocapacitor yarns are characterized by cyclic voltammetry (CV), electrochemical impedance spectroscopy (EIS), chronopotentiometry (CP), and scanning electron microscopy (SEM).

## Results

### Morphology of stretchable organic-inorganic hybrid pseudocapacitor yarn

**Figure 1:**
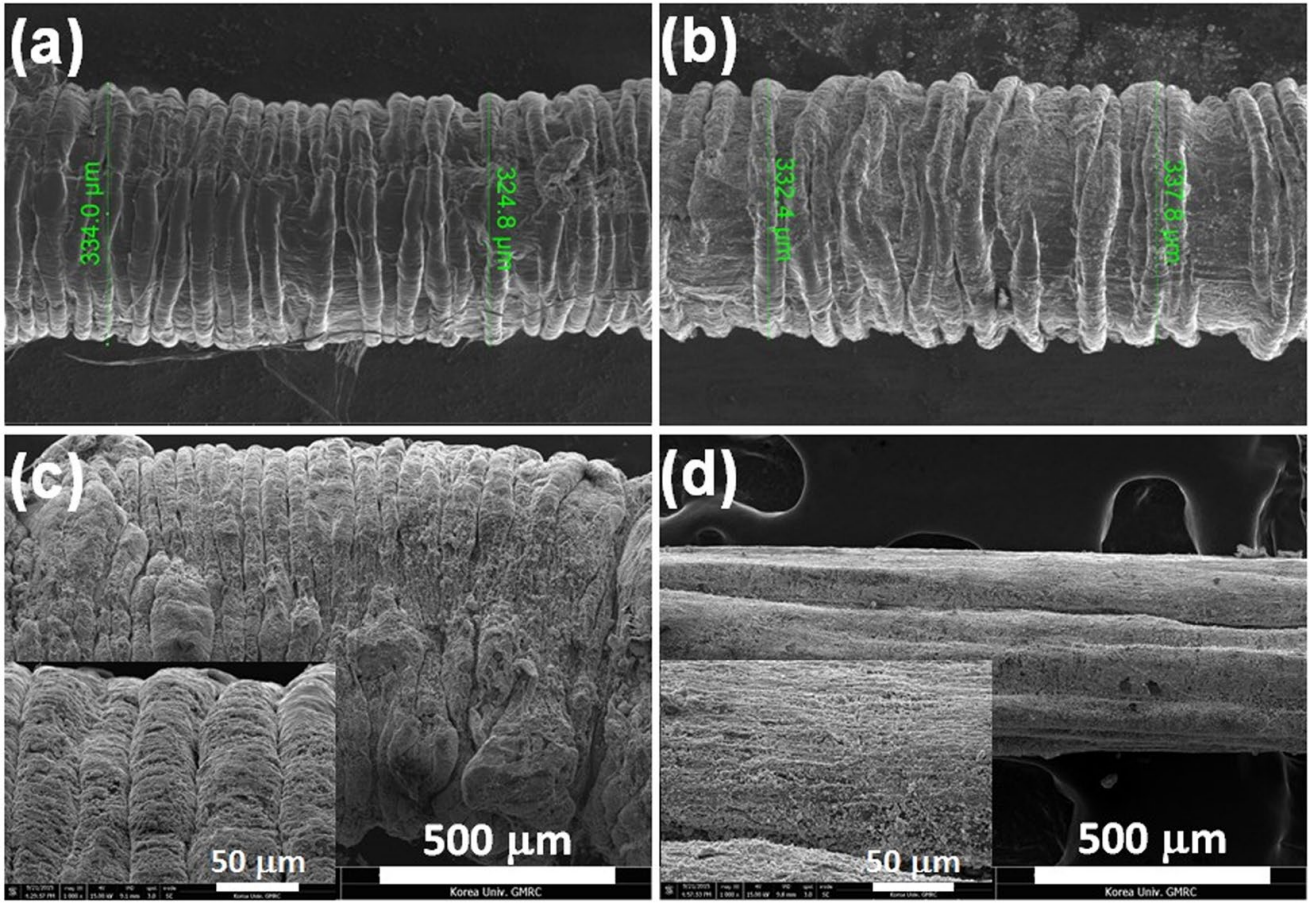
SEM images of stretchable pseudocapacitors: (**a**) as-prepared pseudocapacitor; (**b**) pseudocapacitor after a thousand times of repeated stretching by 200%; (**c**) magnified image of the as-prepared pseudocapacitor showing a wavelength of about 50 μm; (**d**) magnified image of the stretched pseudocapacitor during extension.

Buckling structure, known as a topological pattern that can effectively minimize the spatial constraint of a stretchable device, and one of the most important demands of wearable electronics, is retained after extension of the pseudocapacitor yarn by about 200%. Figure 1a and b show SEM images obtained before and after extending the pseudocapacitor yarn a thousand times. The number of wrinkles per unit length and the diameter of the pseudocapacitor yarn are not appreciably changed after the repeated severe extensions, which verifies the excellent physical reproducibility and ruggedness of the buckling structure. The wavelength of each wrinkle is about 50 μm. Comparison of the SEM image of Fig. 1c with that of Fig. 1d confirms that the stretched surface of the pseudocapacitor is indeed crack free.

### Capacitive characteristics of bare MWCNT-based capacitors

**Figure 2:**
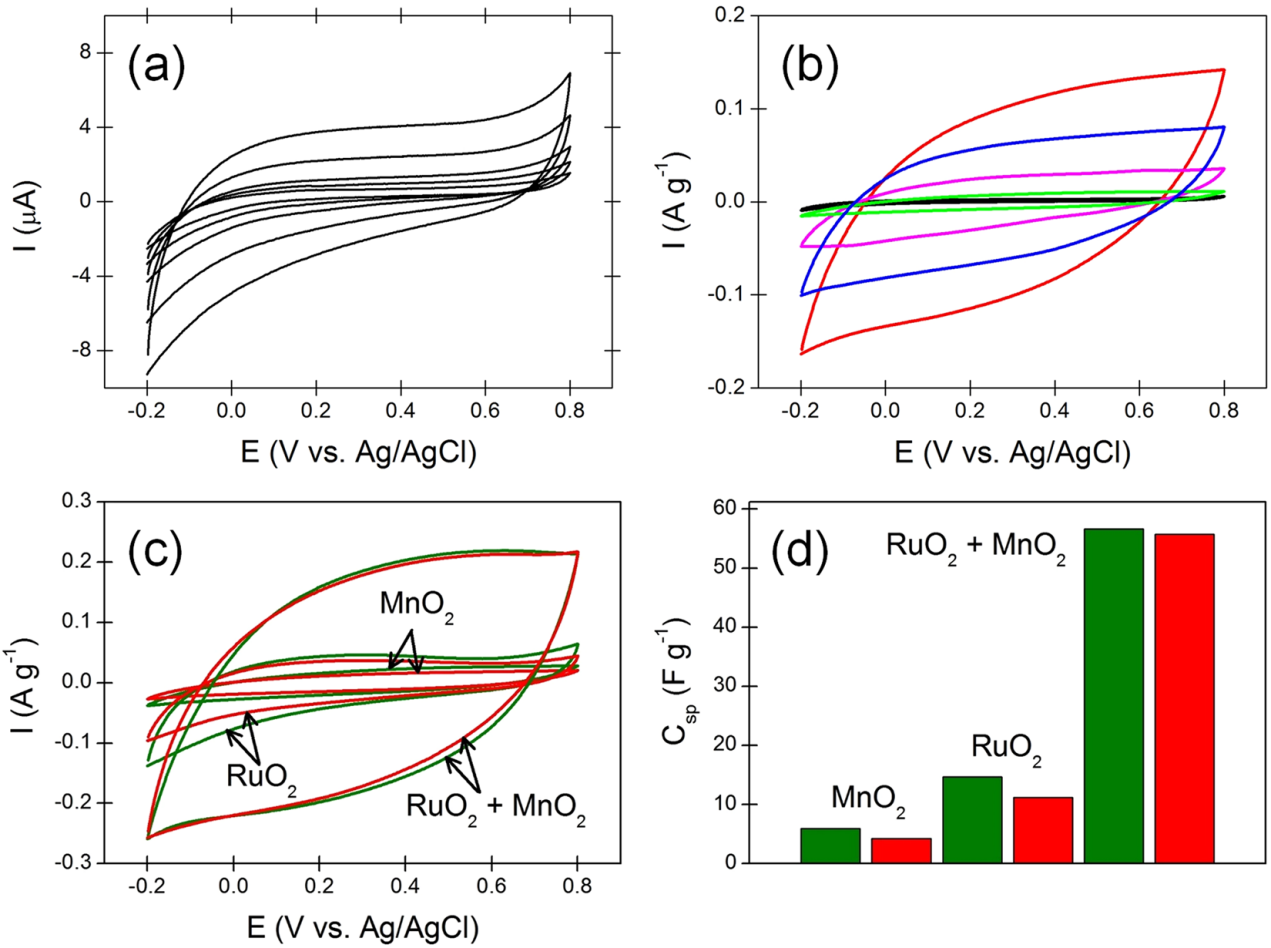
(**a**) Cyclic voltammograms of the yarn type electrochemical capacitor composed of SEBS elastomer and 12-layered MWCNT sheets wrapping the SEBS elastomer at scan rates of 5, 10, 20, 50, and 100 mV s^−1^ in a 1 M Na_2_SO_4_ solution. (**b**) Cyclic voltammograms of variously prepared yarn type pseudocapacitors using different electroactive materials: 10-layered MnO_2_ (green); 10-layered RuO_2_ (pink); 3-layered RuO_2_ + 2-layered MnO_2_ (red). Electrolyte = 1 M Na_2_SO_4_, and scan rate = 5 mV s^−1^. Two additional capacitors, a Pt-free one based on 3-layered RuO_2_ and 2-layered MnO_2_ (blue), and a 12-layered MWCNT-based one with no electroactive materials (black), are also shown for comparison. (**c**) Cyclic voltammograms of variously prepared yarn type pseudocapacitors using different electroactive materials: electrolyte = 1 M Na_2_SO_4_; scan rate = 10 mV s^−1^. Note that green and red solid lines denote graphs obtained before and after stretching the pseudocapacitor yarn, respectively. (**d**) Bar graphs of each pseudocapacitors showing decrease in specific capacitance of the pseudocapacitors: green and red bars denote graphs obtained before and after stretching the pseudocapacitor yarn a thousand times, respectively. Note that 1 layer of Pt powder was employed for each pseudocapacitor to reduce the equivalent series resistance and different layer numbers for different yarns are employed to adjust their stretchability to be around 200%.

The bare MWCNT sheets used in this work basically function as a wrapping material to tightly fix electroactive materials on the SEBS elastomer, and simultaneously act as an electrical conductor that can also partly contribute to the total capacitance. It is known that the many defects naturally formed on carbonaceous materials during various preparation procedures in the form of chemical functional groups can contribute to faradaic capacitance^30^. If the faradaic contribution from the MWCNT sheets was big enough, and even compatible with that from the electroactive NP, it would be meaningful to differentiate the two contributions from each other. It has also been reported that a wire-shaped supercapacitor based on a MWCNT fiber may be possible^2^. To quantitatively determine the contribution from bare MWCNT sheets to the total capacitance, a separately-prepared capacitor yarn was prepared that was composed of SEBS elastomer and 12-layered MWCNT sheets that were absolutely free from any electroactive material. Fig. 2a shows the cyclovoltammetrically recorded capacitive behavior. The measured specific capacitance was 0.133 F g^−1^, which was less than 1% portion of the total capacitance of the hybrid pseudocapacitor yarn. Accordingly, it is concluded that the MWCNT-sheet layers having a complex reticulate structure in the hybrid pseudocapacitor yarn exclusively acts by grabbing electroactive RuO_2_ and MnO_2_ NP while showing negligible capacitance contribution, and also provides a low resistive current path. In this work, mass-based dimension is preferred for the electrical capacity of the pseudocapacitor yarn, rather than the other dimensions such as length-based and area-based ones, because these alternative dimensions strongly depend on how much the pseudocapacitor yarn is stretched. The diameter of SEBS elastomer decreases with extending the elastomer (Supplementary Fig. S1). This means that precise determinations of the cross-sectional area of the SEBS and of the electroactive multilayers that directly cover the SEBS core are in practical terms unavailable.

### Specific capacitances of various hybrid pseudocapacitor yarns

RuO_2_ and MnO_2_ are widely used electroactive materials in electrochemical capacitors, due to their multi-redox potentials that enhance capacitive current flows, outstanding physicochemical stability, and theoretically high specific capacitances that are larger than one thousand farad per gram^31,32^. Although a variety of different methods for the deposition of these electroactive materials are available, physical deposition of the RuO_2_ or MnO_2_ NP denoted by the dry painting in this work was devised in order to avoid serious damage to the stretchability of SEBS elastomer during deposition of the NP on the elastomer. Conventional chemical vapor deposition (CVD) or electrochemical deposition that generally occur in an aqueous medium may provide evenly distributed high quality RuO_2_ or MnO_2_ thin films. However, the deposited thin films prepared by these methods are fragile, and easily cracked during consecutive extension and contraction of the stretchable capacitors. Figure 2b shows cyclic voltammograms of the various pseudocapacitor yarns using different electroactive materials, which indicate that mixing the two electroactive materials in the ratio of RuO_2_:MnO_2_ = 3:2 on the basis of the deposited layer numbers dramatically enhances the specific capacitance. The capacity would be even better if an additional layer of Pt powders aiming to decrease the equivalent series resistance was to be separately deposited. The electrocatalytic effect on oxygen reduction and evolution reactions enhanced by the use of RuO_2_ and MnO_2_ together in lithium-based secondary batteries and methanol fuel cells have been introduced^33–35^. A similar effect can be observed in an electrochemical capacitor having a similar structure to the batteries and fuel cells. From the crystallographic point of view, tunnel size and surface area seem to be the major factors affecting capacity of the MnO_2_-based supercapacitors, and α-MnO_2_, which shows wider tunnel size and larger surface area than the other types of MnO_2_, gives the best electrical capacity^36^. The poor electrical conductivity of MnO_2_ can be complemented by adding a small amount of highly-conductive RuO_2_, and this applies to an electrochemical double-layer capacitor (EDLC)^37^. Figure 2b verifies that in the stretchable pseudocapacitor, as well as in the traditional EDLC as proven previously, MnO_2_ and RuO_2_ complement each other. Cyclic voltammograms of the mixed-layered electrode at different scanning rates are presented in Supplementary Fig. S2 to clarify the rate performance. The specific capacitance of the stretchable pseudocapacitor yarn absolutely decreases by the repeated extensions probably because small portion of the fixed NP are lost during stretching and releasing the yarn. Figure 2c,d show the reduced specific capacitance with the extensions which is confirmed by the cyclic voltammetry of variously prepared yarn type pseudocapacitors. The bar graphs demonstrate that the specific capacitance reduces by approximately 29 ± 5% for the MnO_2_-based pseudocapacitor, 24 ± 5% for the RuO_2_-based pseudocapacitor, and 2 ± 0.5% for the MnO_2_ + RuO_2_-based pseudocapacitor after stretching the yarn a thousand times.

### Electrochemical impedance spectra

**Figure 3:**
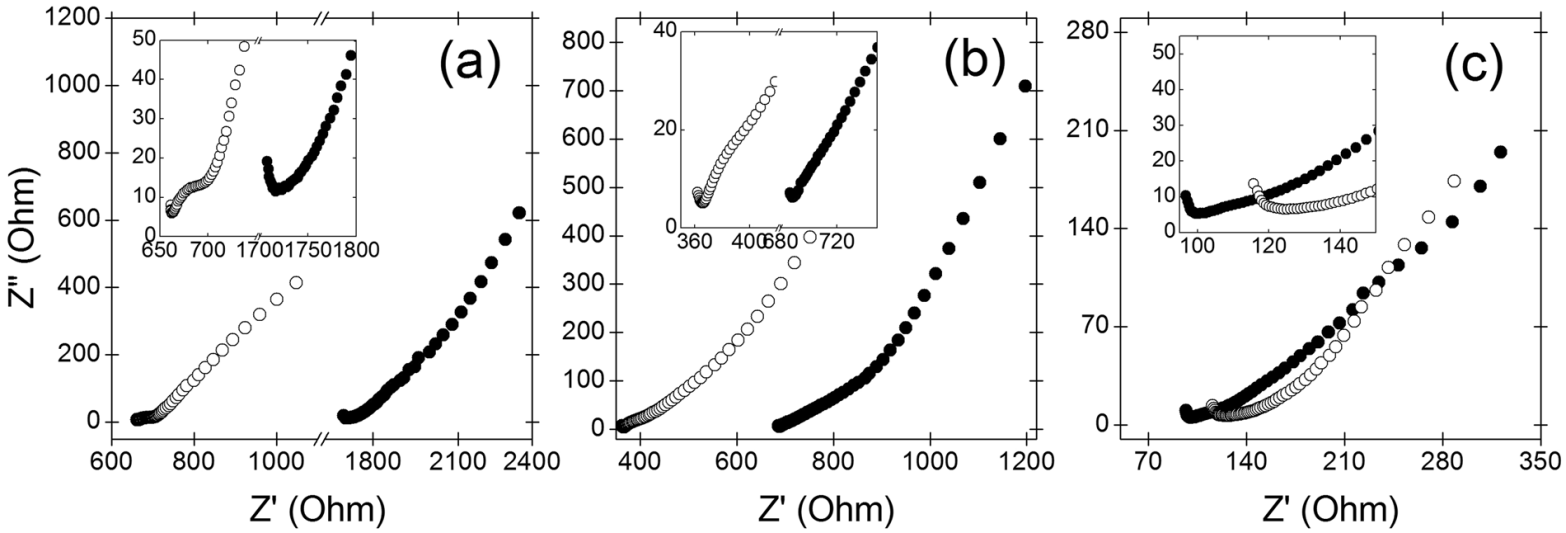
EIS spectra of (**a**) 10-layered MnO_2_-deposited electrode, (**b**) 10-layered RuO_2_-deposited electrode, and (**c**) mixed-layered pseudocapacitive electrode composed of 3-layered RuO_2_ and 2-layered MnO_2_. V_DC_ = 0 V (vs. Ag/AgCl); amplitude = 10 mV; frequency varies from 100 kHz to 100 mHz; electrolyte = 1 M Na_2_SO_4_. Note that open and closed circles denote values obtained before and after stretching the pseudocapacitor yarn, respectively.

Electrochemical double layer capacitors using non-porous electrodes usually show a unique vertical line in the Nyquist plot, which is traditional for a capacitor element. The semicircle diameter of the Nyquist plot represents the sum of the internal resistance of electrode and some other factors such as the electrolyte resistance and contact resistance. Therefore, decreased circle diameter would mean a decrease of the electrode resistance if the other resistances were not variables^38^. However, in a porous electrode-based supercapacitor, such a semicircle is not clearly shown, and no vertical line but a sloped line depending on the pore structure will be observed^30^. Approximately, we can evaluate internal resistances of each porous pseudocapacitors with one another by comparing the real components of the observed impedances in a complex plane because equivalent series resistance is a function of the electrolyte resistance (mostly constant), electrode resistance, and the contact resistance^39^. Figure 3 shows the EIS spectra obtained from the yarn type stretchable pseudocapacitors clearly demonstrate diagonal lines with the typical slope of porous electrode substrates. Indeed, this Warburg like behavior is quite common with porous substrates^30^, and Supplementary Fig. S3 confirms that the stretchable pseudocapacitor yarns in this work have such a highly-porous buckling-structured electroactive layer. The porosity of the electroactive multilayers is around 80% (detailed calculation is available from supporting information). Randomly oriented MWCNT sheets before stretching alter into a well-aligned unidirectional microstructure after stretching. The alignment of electrically conductive MWCNTs along the stretching direction can cause serious decrease in electrical conductivity, especially in the radial direction. Figure 3a,b indicate that the internal series resistances of both MnO_2_-, and RuO_2_-based pseudocapacitors increase after stretching them from 660 Ω to 1710 Ω, and from 365 Ω to 690 Ω, respectively, showing an approximate two-fold increase in series resistance. Interestingly, internal series resistance change before and after stretching is not clearly evident when MnO_2_ and RuO_2_ layers are alternatively deposited on MWCNT sheet substrates, and used as electroactive materials for the pseudocapacitor yarn. More importantly, although the electrical conductivity of RuO_2_ is much larger than that of MnO_2_, the internal series resistances of the mixed-layered pseudocapacitor showing around 120 and 100 Ω before and after stretching (Fig. 3c) are even smaller than those of the RuO_2_-deposited pseudocapacitor.

### Charging and discharging characteristics

**Figure 4:**
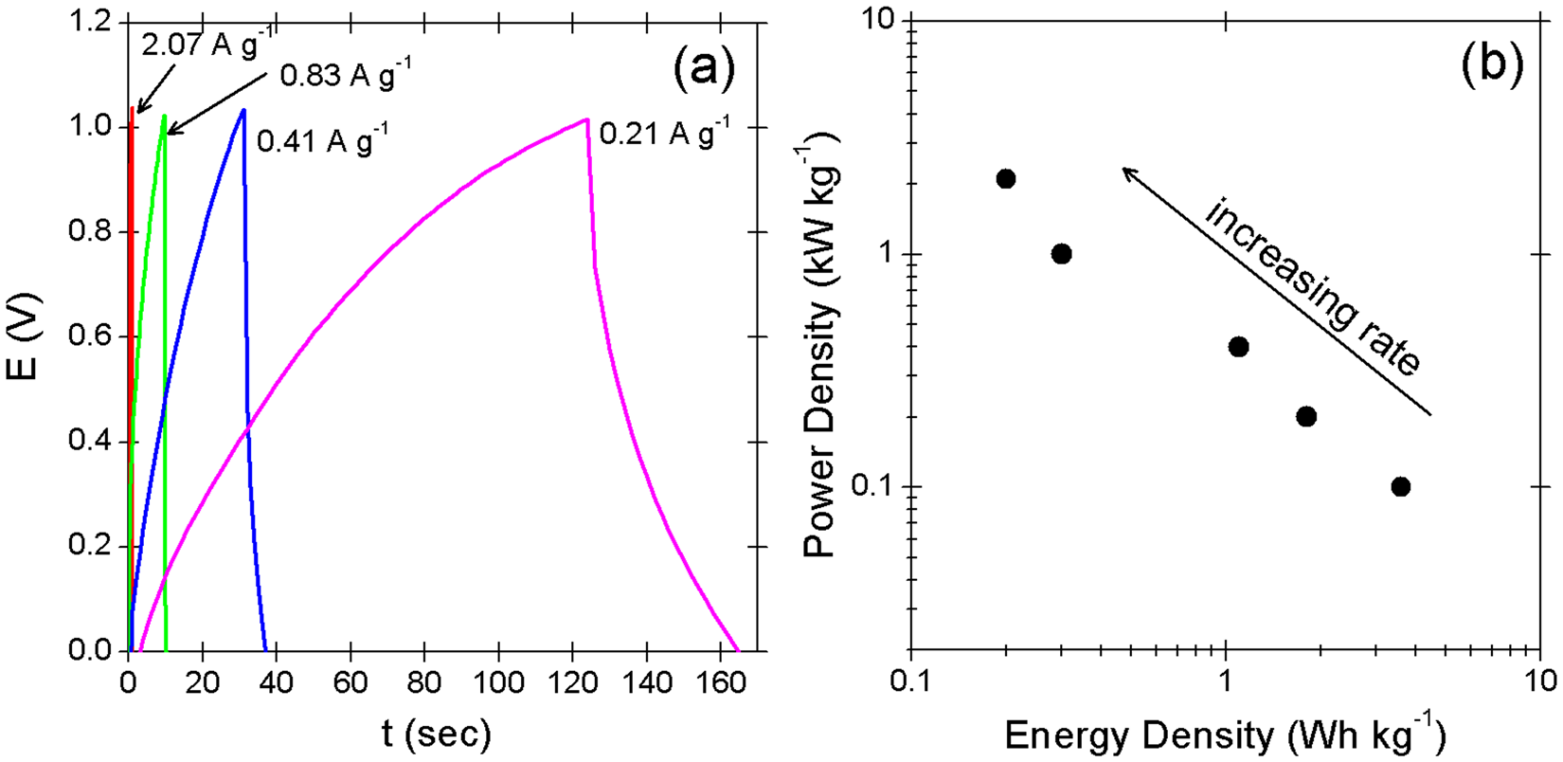
(**a**) Chronopotentiometric responses of the 3-layered RuO_2_ + 2-layered MnO_2_-based pseudocapacitive electrode in 1 M Na_2_SO_4_ solution at various charge rates: reference electrode = Ag/AgCl; counter electrode = Pt wire. (**b**) Ragone plots of the stretchable pseudocapacitor at various charge rates are also shown.

Figure 4a shows that non-linear constant current charge/discharge curves that feature a pseudo-type energy storage mechanism govern the stretchable pseudocapacitor yarn. The specific capacitance at a rate of 0.2 A g^−1^ is approximately 25 F g^−1^. Supporting information 3 provides more detailed descriptions of the computation. Figure 4b shows that the corresponding power density and energy density are inversely proportional to each other in the logarithmic plot.

## Discussion

A variety of different wire electrode-based energy harvesting and energy storage devices have been introduced in recent years, such as dye-sensitized solar cells, supercapacitors, and batteries^10,40,41^. Although some of them will likely be commercially available in the near future^42^, they still suffer from either poor flexibility or poor durability. Therefore, a combination of intrinsically robust inorganic materials with naturally flexible organic materials may give the best results. The problem lies in how to successfully combine them together, without sacrificing too much strength of either organic or inorganic material. In most cases, when both materials are mixed together, the strength of one of them will be totally lost, and unfortunately, the attempt to solve this problem from a macroscopic point of view cannot provide suitable answers. SEBS, as a highly stretchable organic material and an electrical insulator, can have the surface modulated to be electrically conductive by being combined with MWCNT sheets, without serious sacrifice of its stretchability. The MWCNT sheets provide the SEBS with high conductivity. Similarly, the SEBS provides the MWCNT sheets with excellent stretchability, by dispersing the local stress formed during the extension and contraction procedure all the way through the entire MWCNT sheets^43^. Additionally, dry painting of inorganic metal oxides NP on the organic SEBS/MWCNT substrate is a mild and gentle process based on physical adsorption, rather than more radical chemical adsorption, which occasionally results in lethal damage to the organic substrate by any covalent bond formation. Although neither the synergetic effect nor the optimized mixture composition including the optimized layer number of MnO_2_ and RuO_2_ NP in a yarn-type pseudocapacitor are yet fully understood, dry painting is highly versatile, and widely applicable to any NP or other nanostructured materials, especially for yarn-type porous buckling structures.

## Methods

### Preparation of materials

As a conductive substrate, MWCNT sheets were prepared by being withdrawn from the MWCNT forest that was previously grown on a p-type silicon wafer (4-inch-diameter and 17 Ω cm of resistivity) by conventional CVD^44^. A photo image that shows how to prepare the specified MWCNT sheet from the MWCNT forest is available (Supplementary Fig. S4). The general specifications of the MWCNT forest, including the electrical conductivity, specific surface area, tensile strength, and volumetric density, are available elsewhere^45^. Ruthenium Oxide (RuO_2_) and Manganese Oxide (MnO_2_) as electroactive materials purchased from Sigma-Aldrich were first finely ground to have a diameter less than 1 μm, and then deposited on the MWCNTs by the dry painting method. Gel electrolyte was prepared by dissolving a 30 wt% portion of H_3_PO_4_ (Sigma-aldrich) into a 70 wt% portion of polyvinyl alcohol (PVA, Sigma-Aldrich) at 70 °C for 4 hours.

### Electrochemistry

CV diagrams and EIS spectra of yarn type pseudocapacitors in a three-electrode aqueous system composed of Ag/AgCl reference electrode and a Pt wire counter electrode were obtained using a Reference 600 (Gamry Instruments) and Ivium CompactStat 2, respectively. A 1 M Na_2_SO_4_ solution was used as a liquid electrolyte. All electrochemical reagents were used without further purification.

### Fabrication of yarn type stretchable pseudocapacitors

**Figure 5:**
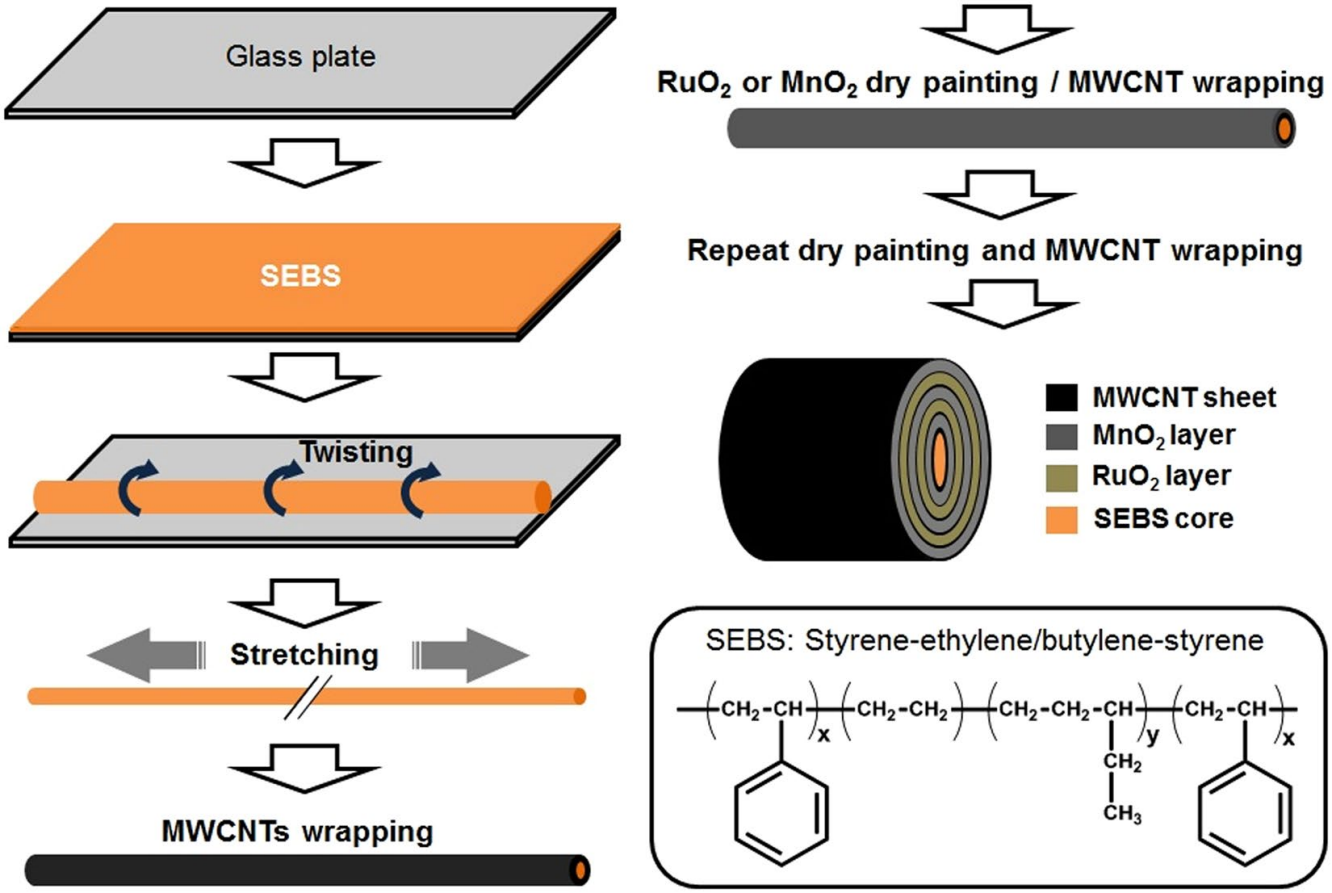
Fabrication process and schematic structure of stretchable pseudocapacitor. Note that the innermost layer from the SEBS core is 3-layered MWCNT sheets, while the other MWCNT layers are 1-layered MWCNT sheet including the outermost one. Note that molecular structure of SEBS is also shown for better understanding.

Figure 5 shows the fabrication procedure of a stretchable pseudocapacitor as follows: a super elastic SEBS core was used as a bottom substrate, and the detailed information about the preparation of SEBS is available^29^. The SEBS elastomer string is first pre-strained by 450%, and then the MWCNT sheet (10 × 1.1 cm) wraps the pre-strained SEBS elastomer using a laboratory made roller. RuO_2_ and MnO_2_ nano particles are alternatively deposited on each MWCNT layer having a unique reticulated structure, which enables any micro and nano particles to be easily captured, and firmly fixed on the cylindrical MWCNT surface layer. Highly stretchable but electrically nonconductive SEBS compensates for the much less stretchable but highly conductive MWCNT layers with each other. The dry painting of the MWCNT-wrapped surface with metallic NP should be sufficient to fully saturate the particle-catching sites of the MWCNT layer, which provides a beneficial effect on suppressing device-to-device variation. Finally, spraying methanol on the stretchable yarn densifies the MWCNT layers, allowing more tight binding of the NP, in order to avoid being liberated from the MWCNT surface. The structural configuration of the stretchable pseudocapacitor depicted in Fig. 5 features alternating metal oxide layers. Three sheets of MWCNT layers are pre-positioned deep inside, i.e., right above the SEBS core, to reduce the equivalent series resistance. After drying, the pseudocapacitor yarn composed of SEBS, MWCNT sheets, RuO_2_, and MnO_2_ NP is gently coated with the gel electrolyte, and the pre-strained yarn is eventually released to assume a buckling structure, which is an ordered periodic one dimensional wavy pattern, as apparently formed in the SEBS elastomer^29^. Two gel electrolyte-coated pseudocapacitor yarns are softly twisted with each other, and the gel electrolyte added again on the twisted yarns for better electrical contact. A video clip that shows the stretching and releasing of the pseudocapacitor yarn is available (Video S1). 200% of stretchability can be obtained with electroactive layers composed of RuO_2_ (3 layers) and MnO_2_ (2 layers).

**Figure 6:**
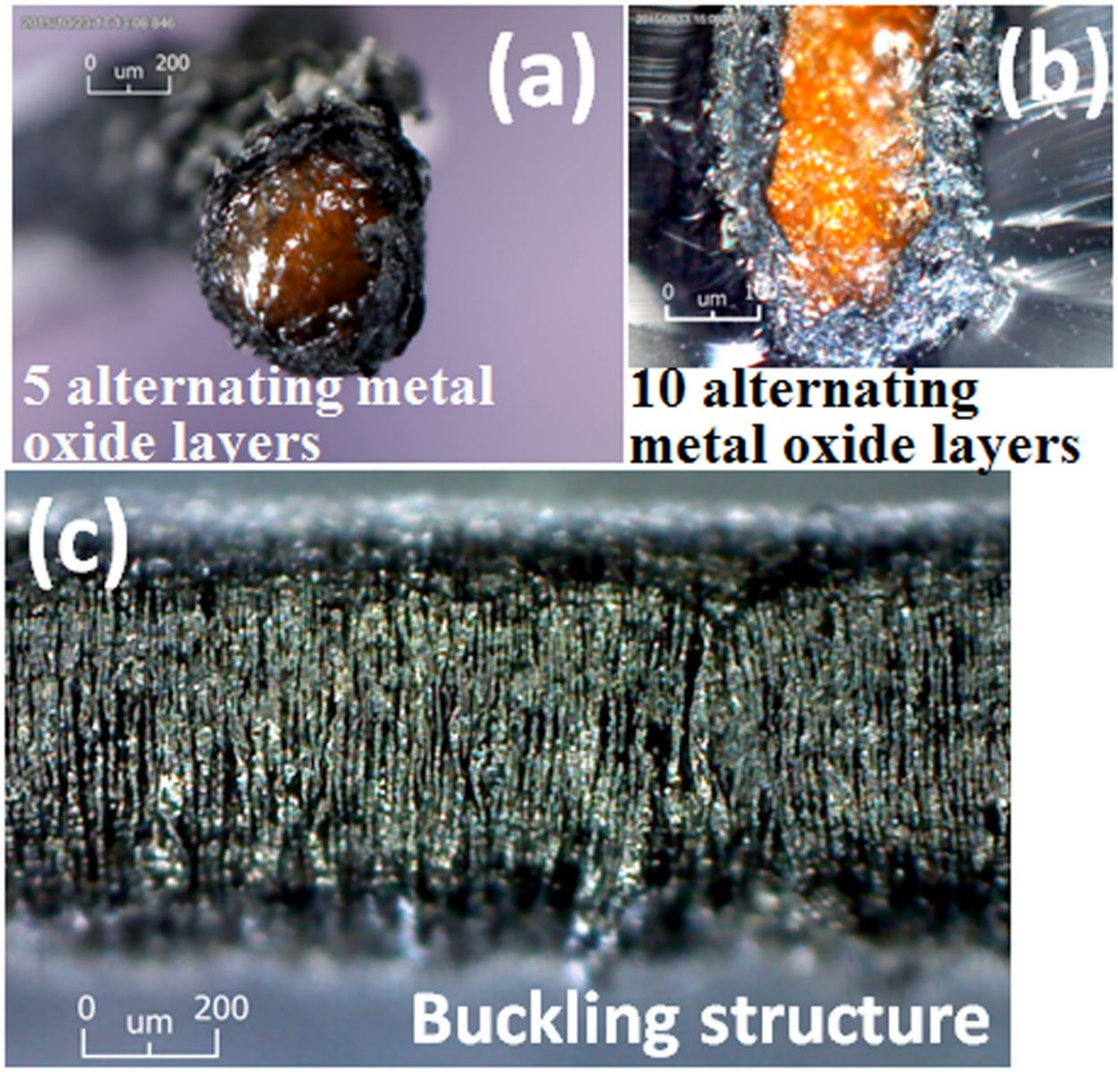
Cross-sectional photo images of the stretchable pseudocapacitor composed of MWCNT sheets, RuO_2_, and MnO_2_ NP with (**a**) five, and (**b**) ten layers of NP, respectively. Note that RuO_2_ layer is the innermost layer for each. Image (**c**) shows the buckling structure of the stretchable pseudocapacitor yarn.

Figure 6a,b demonstrate a clear boundary line between the highly stretchable SEBS core and the surrounding electroactive composite layer that is responsible for the high pseudocapacitance. However, the electroactive layers composed of MWCNT sheets and NP layers, even though they are prepared by a layer-by-layer process, are indistinguishable from each other, indicating that the electroactive composite layers form an electrical single phase (Supplementary Fig. S5), and at least in the outer radial direction, present negligible contact resistance with the relaxed pseudocapacitor yarns (see Fig. 3 for more detailed information). Figure 6c also shows a perfect buckling structure featuring many vertical wrinkles of the stretchable pseudocapacitor yarn.

## Electronic supplementary material

Supplementary video S1

Supplementary information
