# Supplementary material for: Buckling Structured Stretchable Pseudocapacitor Yarn
Source: Sci Rep. 2017 Sep 20;7:12005. doi: 10.1038/s41598-017-12375-7 (PMC5607238; doi:10.1038/s41598-017-12375-7)
Supplement: Supplementary file 2 — Supplementary information [file 41598_2017_12375_MOESM2_ESM.pdf]

Supplementary Information

Buckling Structured Stretchable Pseudocapacitor Yarn

Duck Weon Lee, Jung Han Lee, Nam Ki Min & Joon-Hyung Jin

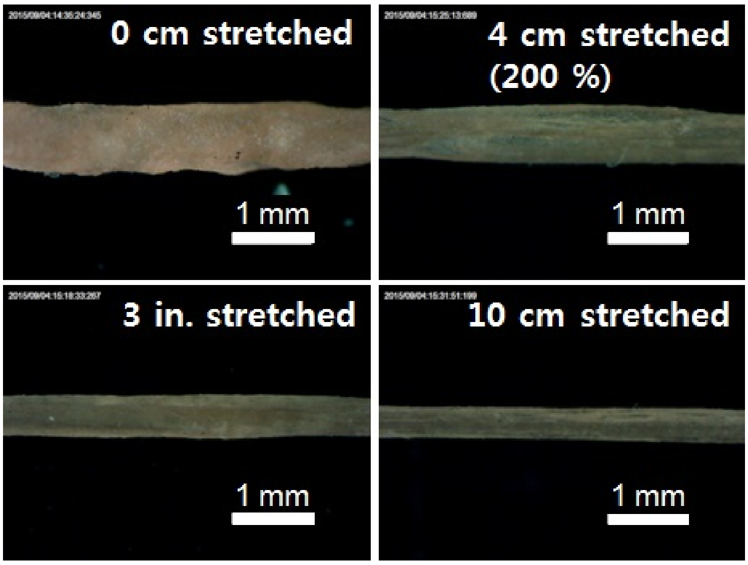

Figure S1. Photo images showing the change in diameter of the SEBS elastomer with various extension.

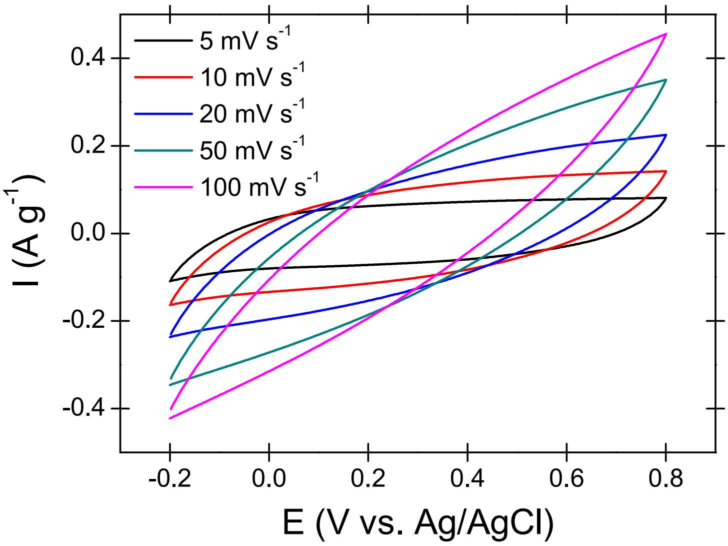

Figure S2. Cyclic voltammograms of a yarn type pseudocapacitor using mixed-layered electroactive materials composed of 3-layered RuO_2 and 2-layered MnO_2 at scan rates of 5, 10, 20, 50, and 100 mV s^{-1} in a 1 M Na_2SO_4 solution.

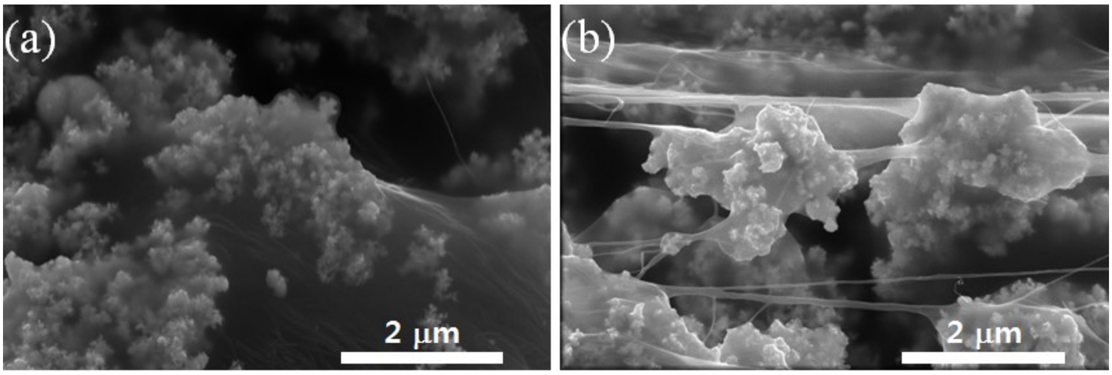

Figure S3. Magnified SEM images of the stretchable pseudocapacitor yarn (a) before, and (b) after stretching the yarn.

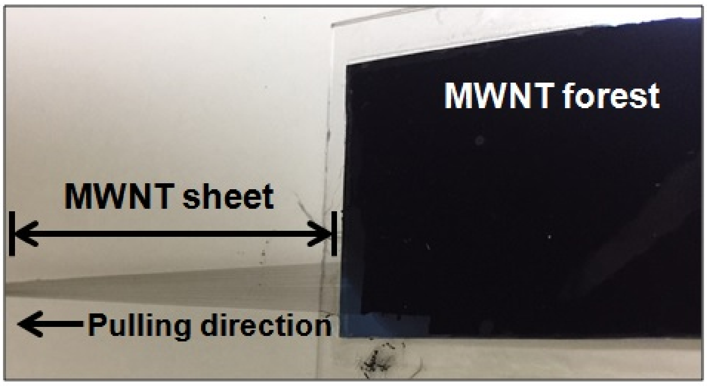

Figure S4. A photo image showing the preparation of the MWNT sheet from the MWNT forest.

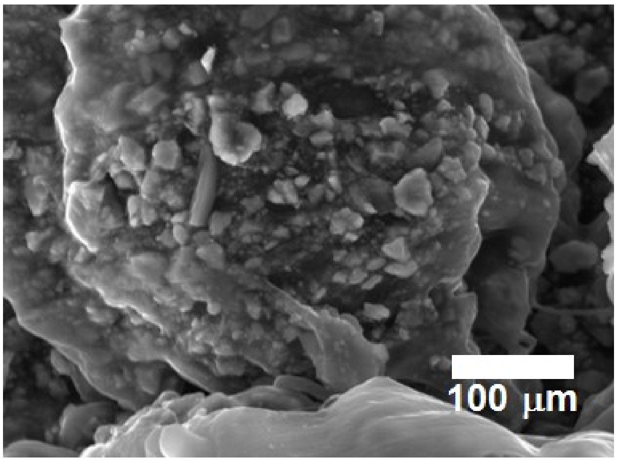

Figure S5. A cross-sectional SEM image of the stretchable pseudocapacitor yarn.

Supplementary
Video S1.avi

Video S1. The video clip shows that repeated stretching and releasing of the pseudocapacitor yarn.

< Calculation >

Specific capacitance in the dimension of farad per gram based on the cyclic voltammogram can be calculated from the following equation:

$$C = \frac{Q}{\Delta V \cdot v \cdot m} \qquad \text{(Equation S1)}$$

where, Q in Coulomb is the area of the closed cyclovoltammetric curve; ΔV in volt is the applied potential window; v in V s^{-1} is the scan rate; and m in gram is the mass of electroactive materials. If the MWNT sheet is the only electroactive material, m can be calculated from the areal mass of the MWNT sheet of 2.7 μg cm^{-2}. Similarly, the specific capacitance can also be calculated from the charge/discharge curve, referring to the following equation:

$$C = \frac{I \cdot \Delta t}{\Delta V \cdot m} \qquad \text{(Equation S2)}$$

where, I represents the applied constant current in ampere, and Δt is the discharging time in second. ΔV and m are the same as described above. Additionally, the specific energy and power are calculated by the following equations, respectively.[1,2]

$$E = \frac{C \cdot \Delta V^2}{2} \qquad \text{(Equation S3)}$$

$$P = \frac{E}{\Delta t} \qquad \text{(Equation S4)}$$

Volumetric void fraction denoted by collective porosity in the text is defined by the following equation with assuming that: the stretchable pseudocapacitor yarn is a cylindrical structure with a smooth surface; the volume of NPs on each MWNT sheet layer is the same as each other; and the volume of the SEBS core is invariable.

$$\text{Volumetric void fraction} = 1 - \frac{\text{filled volume of the electroactive layers}}{\text{total volume of the electroactive layers}}$$

The denominator of the equation is determined by subtracting the volume of the SEBS core from that of the pseudocapacitor yarn. The numerator could be determined, if the densities of each NPs and MWNT sheet were available. Densities of RuO_2, MnO_2, and the densified MWNT sheet are 6.97, 5.026, and 0.8 g cm^{-3}, respectively.[3] Note that the calculation uses an averaged density of the electroactive NPs.

REFERENCES

1 Gomez, J. & Kalu, E. E. High-performance binder-free Co-Mn composite oxide supercapacitor electrode. *J Power Sources* **230**, 218-224, doi:10.1016/j.jpowsour.2012.12.069 (2013).

2 Conway, B. E. *Electrochemical supercapacitors : scientific fundamentals and technological applications*. (Plenum Press, 1999).

3 Zhang, M., Atkinson, K. R. & Baughman, R. H. Multifunctional carbon nanotube yarns by downsizing an ancient technology. *Science* **306**, 1358-1361, doi:DOI 10.1126/science.1104276 (2004).
